# 

**Published:** 1894-10

**Authors:** 


### CONTENTS.

ORIGINAL ARTICLES—

A Report of Some Rare Surgical Lesions Connected with the Liver. By John A. Wyeth, M.D., New York .......... 523

The Slaughter of the Innocents. By E. Van Goidtsnoven, M.D., Atlanta, Ga .......... 531

The Treatment of Pneumonia in Children. By Frank S. Parsons, M.D., Philadelphia, Pa .......... 537

Cold Water as an Antipyretic .......... 543

SOCIETY REPORTS—

Tri-State Medical Society of Alabama, Georgia, and Tennessee. Sixth Annual Session .......... 544

BOOK REVIEWS—

A Treatise on the Principles and Practice of Medicine. Designed for the Use of Practitioners and Students of Medicine. By Austin Flint, New York City .......... 561

Hare's Text-Book of Practical Therapeutics: A Text Book of Practical Therapeutics; With Especial Reference to the Application of Remedial Measures to Disease and their Employment upon a Rational Basis. By Hobart Amory Hare, M.D., Philadelphia, Pa.... 562

SELECTIONS AND ABSTRACTS—

Treatment of Fistula in Ano. By Geo. J. Cook, M.D., Indianapolis, Ind...... 562

EDITORIAL—

Notice ...... 566

Private Patients in Grady Hospital...... 566

Dr. Oliver Wendell Holmes...... 568

EDITORIAL NOTES...... 569-570

PRESCRIPTION DEPARTMENT...... 572, 573

Chronic Rheumatism—To Allay the Craving for Alcohol—Cholera Infantum—Eczema of Scalp—Excoriation in Children—Diuretic in Cardiac and Renal Dropsy—Periostitis—Fetid Diarrhea in Children—Whooping Cough—Dipsomania.

SPECIAL NOTES ...... 574, 576, 578, 580

